# Assessing Patient-Specific Microwave Breast Imaging in Clinical Case Studies

**DOI:** 10.3390/s21238048

**Published:** 2021-12-01

**Authors:** Declan O’Loughlin, Muhammad Adnan Elahi, Benjamin R. Lavoie, Elise C. Fear, Martin O’Halloran

**Affiliations:** 1 Electronic and Electrical Engineering, Trinity College Dublin, D02 PN40 Dublin, Ireland; 2 Electrical and Electronic Engineering, National University of Ireland Galway, H91 TK33 Galway, Ireland; adnan.elahi@nuigalway.ie (M.A.E.); martin.ohalloran@nuigalway.ie (M.O’H.); 3 Electrical and Computer Engineering, University of Calgary, Calgary, AB T2N 1N4, Canada; brlavoie@ucalgary.ca (B.R.L.); fear@ucalgary.ca (E.C.F.)

**Keywords:** microwave breast imaging, imaging algorithms, patient images, patient-specific imaging

## Abstract

Microwave breast imaging has seen increasing use in clinical investigations in the past decade with over eight systems having being trialled with patients. The majority of systems use radar-based algorithms to reconstruct the image shown to the clinician which requires an estimate of the dielectric properties of the breast to synthetically focus signals to reconstruct the image. Both simulated and experimental studies have shown that, even in simplified scenarios, misestimation of the dielectric properties can impair both the image quality and tumour detection. Many methods have been proposed to address the issue of the estimation of dielectric properties, but few have been tested with patient images. In this work, a leading approach for dielectric properties estimation based on the computation of many candidate images for microwave breast imaging is analysed with patient images for the first time. Using five clinical case studies of both healthy breasts and breasts with abnormalities, the advantages and disadvantages of computational patient-specific microwave breast image reconstruction are highlighted.

## 1. Introduction

In recent years, more than eight microwave breast imaging systems have been used with patients for some ongoing trials including almost 1000 patients [1,2,3,4,5,6,7]. Two systems are being developed commercially, the MARIA system by Micrima, Bristol, the UK and also the Wavelia system by Microwave Vision Group, Paris, France, both of which have been used in clinical investigations [8,9]. Additionally, a number of research groups have published patient images, including from Dartmouth University, the University of Calgary and Hiroshima University among others [5,10,11,12,13,14].

In addition to patient trials, both analytical and experimental investigations of the fundamentals of microwave breast imaging have also continued, examining factors such as antenna layout, artefact removal, imaging algorithm selection, prior information integration, multi-modality imaging, contrast agents and phantom development [15,16,17,18,19,20,21,22,23,24,25,26,27,28]. In the past two years, open-source imaging tools such as the MERIT toolbox, introductory textbooks such as that published by Nikolova and now open-source experimental data have been made available to the microwave imaging community [1,29,30].

Many of the challenges faced when imaging patients identified from the clinical studies to date can be categorised in four broad areas:

1. Inefficient coupling of energy into the breast [31];
2. Imaging domain changes during acquisition [31,32,33];
3. Intrapatient variation due to the menstrual cycle, hormonal changes or weight differences [33];
4. Interpatient variation in breast size, shape and composition [10,18,21].

These four challenges can have a large impact on image quality: if microwave energy is inefficiently coupled into the breast (challenge 1), the tumour response in the scattered signals may be very small or even below the noise floor. Practical solutions employed by the operational systems to overcome these challenges are discussed in this section, including the aspects of these challenges yet to be resolved.

The patient interface design can help address challenge 1. The design of biocompatible coupling media which are stable over time, have appropriate dielectric properties and are cheap and easy to replace between patients [34,35] improves the quality of the total field recording acquired. Coupling media are often designed with lossy dielectric properties to reduce reflections from the tank boundaries and any other unwanted reflections which may hamper reconstruction [35]. Later generations of MARIA also include automated quality checking to ensure efficient coupling at all antennas [36].

The acquisition hardware can also help address challenge 1 [37]. For example, the latest generation of TSAR was designed to increase the penetration of energy into the breast by automatically repositioning the antennas perpendicular to the breast surface [38]. Other types of radome design and acquisition hardware have also been proposed and are being tested experimentally, which could help ensure efficient coupling of microwave energy into the breast. For example, a multi-faceted metal chamber in the general shape of a hemiellipsoid with magnetic half-loop probes has been presented [39]. The irregular shape can improve the reconstruction quality and the chamber is designed to maximise penetration of microwave energy into the breast [39].

The imaging domain is also subject to change during the scan (challenge 2): the breast can move due to patient breathing or discomfort, or blood flow or temperature changes may occur in the living breast tissue [31,32]. Shorter acquisition times can help minimise the effects of these changes during acquisition, and later generations of MARIA were designed to acquire the complete scan in under one minute [9,36]. As with challenge 1, improved coupling medium design can help immobilise the breast during the scan [34].

Studies using TSAR have looked at the repeatability of the scans (challenges 2 and 3), highlighting the differences that can occur between patient scans and proposing metrics which can be used to quantify these differences independently [21]. Studies with healthy volunteers from McGill University have also evaluated the effects of patient position and movement during the scan and analysed the effect of the menstrual cycle and other natural changes on the images [33].

As microwave imaging is used with more diverse study populations, challenge 4 is becoming increasingly important [40]. Patient interfaces, acquisition hardware and coupling media are now being designed to accommodate more breast sizes and shapes [9]. In terms of interpatient variation in breast tissue composition, many studies have identified that the breast composition can affect microwave image quality [41,42,43,44,45,46,47,48]. However, most published patient imaging studies do not adjust the breast composition assumptions on a patient-by-patient basis [9,11,12,33,49]. Patient imaging studies from the University of Calgary did identify interpatient variance of breast tissue composition as an important parameter for imaging [11]. Subsequent studies have considered computational patient-specific beamforming to account for interpatient variance [48,50]. This work expands on these studies to form a comprehensive study of the potential benefits in terms of the sensitivity of radar-based imaging.

A number of complexities exist when interpreting the results of clinical studies using microwave breast imaging:

1. Radar-based images are typically compared to images from other modalities such as mammography, ultrasound or magnetic resonance imaging which are acquired from different orientations;
2. Images from other modalities exploit different properties of human tissues (such as X-ray attenuation using mammography), which makes the comparison of breast structures between images from different modalities difficult. For example, microcalcifications are very prominent in mammograms but may not be visible at all in radar-based images;
3. The breast often contains multiple regions of interest which would be expected to be visible in the microwave breast image and it is not clear what the optimal radar-based image should look like in these cases;
4. The true dielectric properties of the breast are not known quantitatively, only qualitative assessments of breast density from mammography are known, which measure the proportions and distribution of glandular tissues but not the dielectric properties.

In this work, the performance of a computational patient-specific imaging algorithm is analysed in five clinical case studies from the TSAR system developed by the University of Calgary [11]. These clinical case studies were first published in [11] using fixed-value reconstruction permittivity estimation with the DAS beamformer, in a preliminary study of a parameter search reconstruction permittivity estimation algorithm in [48], and used in a beamformer comparative study in [19]. In this work, these five clinical case studies are used to investigate the potential of patient-specific beamforming to improve the sensitivity without impairing the specificity of radar-based breast imaging algorithms. Additionally, the challenges of designing and analysing clinical trial results are identified.

The remainder of this paper is structured as follows: the patient population, clinical procedure and imaging algorithm are described in Section 2, the results are presented in detail in Section 3 and Section 4 concludes the paper.

## 2. Methodology

In this section, the patient population, clinical procedure, imaging algorithm and analysis techniques are outlined. All patient images were reconstructed using scattered data acquired from the TSAR system developed at the University of Calgary and were originally published in [11]. The operational system itself is described fully in [51] and the next generation system is described in detail in [15]. Other patient imaging studies using these data, such as [11,19,48], have been comprehensively reviewed and compared to the literature in [5].

All patients were recruited from the Breast Health Clinic, Foothills Medical Centre, Calgary, AB, Canada and provided written informed consent to participate in the study (E-22121 approved by the Conjoint Health Research Ethics Board, University of Calgary, AB, Canada). Patients with a breast size of B or C cup, with suspicious areas in the breast not located in the axilla, were considered for inclusion in the study. Only patients who were eligible for magnetic resonance imaging (for example, no metallic implants) were included.

Prior to radar-based imaging with TSAR, the ipsilateral breast was scanned using magnetic resonance imaging no more than four days before the TSAR scan, except for one patient for which it was twelve days earlier. The magnetic resonance image was acquired using a 1.5 T scanner with breast coils, and both pre-contrast and subtracted images were used as part of the clinical history of the patient. Additional clinical information such as recent mammograms, ultrasound studies, image reports, biopsy results and pathology report were also assessed to provide a complete clinical history of the patient.

Five clinical case studies are analysed in detail in this work, and the relevant clinical information for each case is summarised in Table 1. The case studies cover three BI-RADS density categories: scattered heterogeneous (category B) in the case of Patient 3, heterogeneous (category C) in cases 1, 4 and 5, and extremely dense for case 2. Cases 1–3 are called Group A and are characterised by having a clearly identified disease. Cases 4 and 5 are called Group B in the original study in [11], and multiple suspicious lesions were identified from the complete clinical history.

For each patient scan, the patient lay prone on the examination table with the breast pendant through an opening. The breast was immersed in canola oil which has relative permittivity of 2.5 and conductivity of 0.04 S $m^{-1}$ up to 12 GHz. A single BAVA-D, described in [52], was used to acquire the scattered signals from up to 200 antenna locations around the breast. The antenna can move in the sagittal direction (vertically) and the tank and antenna rotate to illuminate from all angles in the coronal plane (horizontal). In addition to the microwave scan, an integrated laser ranging system and optical camera are used to estimate the breast surface.

Scattered data were acquired between 50 MHz and 15 GHz and three measurements were averaged to improve the noise floor. A second scan using the same antenna locations but without the patient breast was also performed. This scan without the patient breast was used for calibration. After calibration, the scattered data were shaped with a differentiated Gaussian pulse with centre frequency of 4 GHz and full width at half-maximum (FWHM) of 6.3 GHz. A phase shift was also introduced to compensate for the antenna aperture location.

The neighbourhood-based skin subtraction artefact removal algorithm was employed to isolate reflections from the breast interior [53]. TSAR includes a laser ranging system mounted on the same positioning arm as the antenna, which measures the distance to the skin for each antenna location. After artefact removal, the signals are synthetically focused and the image is reconstructed using the DAS beamformer. The imaging domain is divided into two regions:

1. The coupling medium with a known relative permittivity of $\varepsilon_{r}^{\mathrm{cm}}=2.5$;
2. The breast interior with an assumed reconstruction permittivity $\varepsilon_{r}^{\prime }$.

In this work, the assumed reconstruction permittivity of the breast interior was swept in the range $4<\varepsilon_{r}^{\prime }<16$, while the assumed permittivity of the coupling medium was held constant at $\varepsilon_{r}^{\mathrm{cm}}=2.5$. Previous work has also examined the effect of varying both parameters [48]. This permittivity range includes the values used in the original study, $\varepsilon_{r}^{\prime }=9$ in [11], and by MARIA, $\varepsilon_{r}^{\prime }=10$ in [9].

For each reconstruction permittivity value $\varepsilon_{r}^{\prime }$ in the range, a full three-dimensional delay-and-sum (DAS) image was reconstructed using the open-source MERIT toolbox [29]. Using laser data from the TSAR system, the imaging domain was confined to the area within the breast. A gradient-based metric was applied to each image, where the metric value, $\Phi_{\mathrm{DMA}}^{G}$, is calculated as follows:

(1) $$\Phi_{\mathrm{DMA}}^{G}=\langle \max_{D}\left|I_{D}^{D})\right|\rangle \;\forall D\in \left\{X,Y,Z\right\}$$

where:

$\langle \;\cdot \;\rangle$ is the average of the contents;
- $I_{D}^{D}$ are the first order differences along each dimension *D* of the image.

This metric and similar approximations to the three-dimensional gradient have been used in both autofocus and imaging applications to select images based on sharpness, and the choice of metric and theoretical understanding of the metric are further explained in [46]. In the following results, the metric values for each image and each case are shown and peaks of the metric are used to identify the images to analyse.

Additionally, each image is examined quantitatively using:

- The FWHM of the image, defined for each dimension (*D*) as:
  (2) FWHMD(I)=mindD12−+mindD12+
  where dD12− and dD12+ are sets of distances to the points along the *D* axis where the magnitude of the image is less than half the maximum magnitude in the positive and negative directions, respectively:
  (3) dD12−=d|I(rmax−dD^)=0.5I(rmax)|∀0<d<dmax
  and
  (4) dD12+=d|I(rmax+dD^)=0.5I(rmax)|∀0<d<dmax
  where $r^{\max}$ is the location of the maximum energy in the image.
- The signal-to-clutter ratio (SCR), defined in this work as:
  (5) $$\mathrm{SCR}\left(I\right)=\frac{I(r^{\max})}{I(r^{\mathrm{clut}})}$$
  where $r^{\max}$ is the maximum intensity in the clutter region, defined as the area greater than twice the FWHM away from $r^{\max}$.
- Similarly, the signal-to-mean ratio (SMR), defined in this work as:
  (6) $$\mathrm{SMR}\left(I\right)=\frac{I(r^{\max})}{\langle I^{\mathrm{clut}}\rangle }$$
  where $I^{\mathrm{clut}}$ is the set of points in the clutter region.

## 3. Results

**Figure 1:**
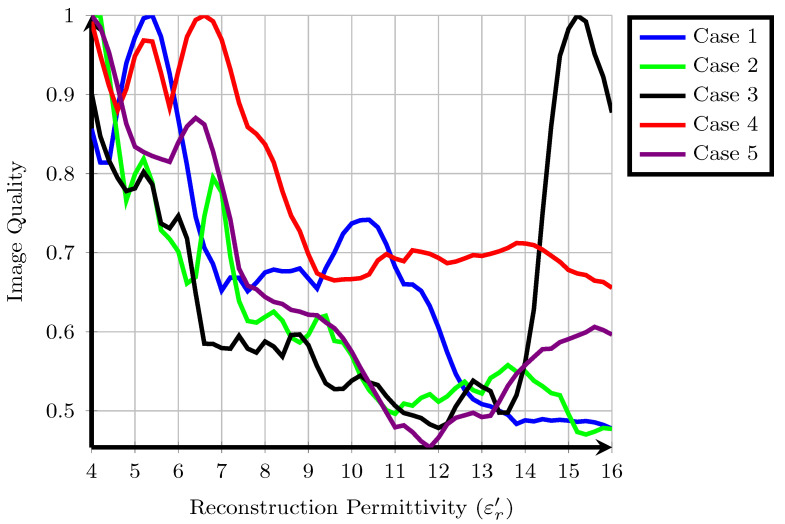
A suitable cost function applied to five clinical case studies from the TSAR system, values are normalised so that the maximum amplitude is equal to one.

The cost function values, $\Phi_{\mathrm{DMA}}^{G}$, for each of the five patients are shown in Figure 1. Higher cost function values are typically associated with images containing one sharp response such as a tumour and lower overall energy. Some overall trends are visible when all patients are considered together. Firstly, with the exception of Patient 3, the majority of local maxima of the fitness occur for approximately $\varepsilon_{r}^{\prime }\leq 10$. Typical values from the literature for the reconstruction permittivity are also in this range, suggesting that higher values represent signals from long propagation paths with higher loss and less energy. Similarly, the fitness of images reconstructed with a lower reconstruction permittivity tends to be higher than those reconstructed with a higher reconstruction permittivity: in cases such as Patient 2, a downward trend in fitness is visible as the reconstruction permittivity increases. This downward trend does not necessarily suggest that all images at lower reconstruction permittivities are of higher quality, as previous experimental studies have shown that the properties of underestimated images may resemble tumour images in many cases, as in [46].

Secondly, the fitness for many patients shows multiple local maxima. In some cases, such as Patient 1, the local maximum at $\varepsilon_{r}^{\prime }=10.4$ is 25% lower than the global maximum at $\varepsilon_{r}^{\prime }=5.4$. In other cases, such as Patient 4, the local maximum at $\varepsilon_{r}^{\prime }=5.4$ is only 5% lower than the global maximum at $\varepsilon_{r}^{\prime }=6.6$. A similar trend was observed using a different cost function in previous work [48] and also in other experimental work [18], where a range of reconstruction permittivity values can result in images which are visually very similar. However, in some cases, such as Patient 1, images at the different extrema are substantially different, whereas in Patient 4, the locations of the regions of high intensity are the same.

Finally, the images analysed in the original study were reconstructed at $\varepsilon_{r}^{\prime }=9$ in all cases. In these five patient cases, the original images are not rewarded highly by the cost function, the original images reconstructed at $\varepsilon_{r}^{\prime }=9$ are not local or global maxima. However, in many cases, such as Patient 1, Patient 2, Patient 4 and Patient 5, an image with similar energy distribution was highly rewarded at a nearby reconstruction permittivity value. The results of each individual patient are discussed in detail in the following subsections.

### 3.1. Metaplastic Carcinoma (Case 1)

The right breast of 53-year-old Patient 1 was scanned. Three regions of interest in the breast were identified from mammography, the magnetic resonance image and the original study [11], which are described here:

- $R_{1}^{1}$ corresponding to a 10 mm mass detected at the 4 o’clock radian using mammography. The same mass was detected at the 5 o’clock radian using magnetic resonance imaging. Postsurgical pathology indicated that the mass in $R_{1}^{1}$ was a grade II/III metaplastic carcinoma;
- $R_{2}^{1}$ is a possibly benign lesion detected at the 7 o’clock radian through magnetic resonance imaging but not mentioned in the pathology report;
- $R_{3}^{1}$ refers to a cluster of glandular tissues located at the 11 o’clock radian.

**Figure 2:**
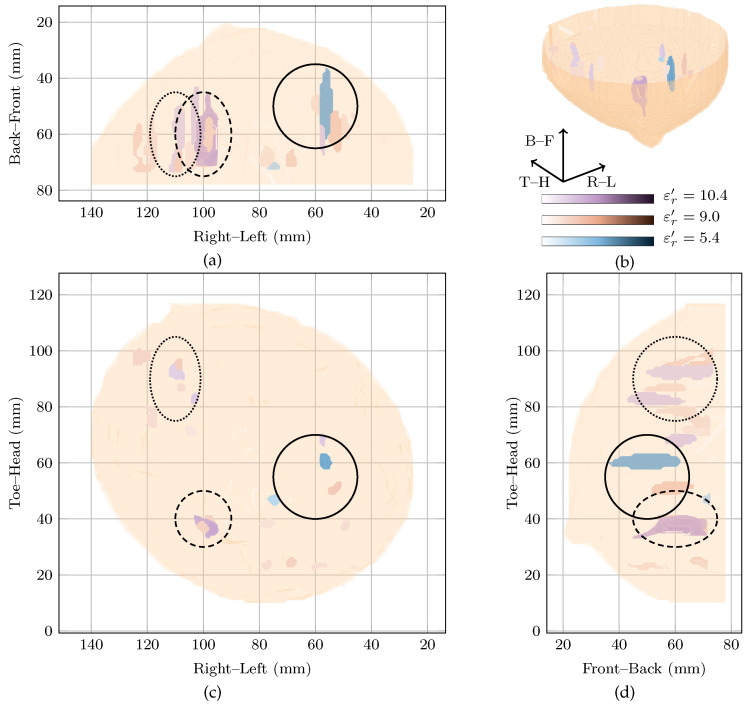
Images of high fitness and the image from the original study of Patient 1 compared in three dimensions in (**b**). Shown also are the coronal in (**d**), sagittal in (**c**) and axial in (**a**) slices.

The approximate locations of $R_{1}^{1}$, $R_{2}^{1}$ and $R_{3}^{1}$ are shown in Figure 2 by the solid, dashed and dotted lines.

Two images are highly rewarded by the cost function in Figure 1 for this patient: a global maximum at $\varepsilon_{r}^{\prime }=5.4$ and a local maximum at $\varepsilon_{r}^{\prime }=10.2$. To compare the images, the regions of high intensity from both images are shown in Figure 2. Regions from the image at the global maximum at $\varepsilon_{r}^{\prime }=5.4$ are shown in purple whereas those from the image at the local maximum at $\varepsilon_{r}^{\prime }=10.4$ are shown in blue. The image reconstructed at $\varepsilon_{r}^{\prime }=9.0$ is also shown in red in Figure 2.

Considering the image at the global maximum at $\varepsilon_{r}^{\prime }=5.4$ alone (blue in Figure 2), a large response is visible near $R_{1}^{1}$ corresponding to the malignant tumour. The response is elongated along the sagittal axis (FWHM of 41 mm as can be seen in Figure 2), but is smaller along the vertical and axial axes with an average FWHM of 9 mm in the coronal plane. The other response in the image at the global maximum is 6 dB lower in amplitude, considerably smaller (less than 10 mm maximum FWHM) and located at the edge of the imaging domain.

The image at the local maximum at $\varepsilon_{r}^{\prime }=10.4$ (shown in red in Figure 2) contains three main groups of responses in the three regions of interest, $R_{1}^{1}$, $R_{2}^{1}$ and $R_{3}^{1}$. The response with the highest energy is located close to $R_{2}^{1}$ and corresponds to a possible benign lesion. This maximum response has an average FWHM of 8 mm in the coronal plane. The other responses in the image are at least 6 dB lower in amplitude than the maximum response. The next two responses are located in $R_{3}^{1}$ and $R_{2}^{1}$, respectively, corresponding to a fibroglandular cluster and the malignant lesion. Similar to the image at the global maximum, the responses are elongated along the sagittal axis, with FWHM of 23, 38 and 25 mm in this direction for the first three responses, respectively.

Comparing the three images, the maximum amplitude of the image reconstructed at $\varepsilon_{r}^{\prime }=10.4$ is 1.6 dB lower than the image reconstructed at $\varepsilon_{r}^{\prime }=5.4$, the global maximum. Both images show energy in $R_{1}^{1}$, the malignant tumour, and the response in $R_{1}^{1}$ at the global maximum is the response of maximum amplitude in all images.

The two images rewarded by the cost function at $\varepsilon_{r}^{\prime }=5.4$ and $\varepsilon_{r}^{\prime }=10.4$ are quantitatively compared to the image in the original study [11], in Table 2. All values are normalised such that the maximum is 0 dB. The image from the original study [11], showed energy in all three regions of interest, with the maximum energy corresponding to the malignant lesion in $R_{1}^{1}$. The maximum energy in the other two regions of interest—$R_{2}^{1}$ and $R_{3}^{1}$—was 1.4 dB and 0.6 dB below the maximum energy. The maximum energy in $R_{1}^{1}$ is 5.48 dB below the global maximum image reconstructed at $\varepsilon_{r}^{\prime }=5.4$.

### 3.2. Fibroadenolipoma (Case 2)

Case 2 involved a 64-year-old woman with a fibroadenolipoma in the lower inner quadrant of her left breast. The mammography report notes the breast tissue is extremely dense (BI-RADS category D). Fibroadenolipomas (also known as hamartomas) are typically benign masses containing an admixture of ducts, lobules, fibrous stroma and adipose tissues in varying proportions. The contrast between the fibroadenolipoma and the surrounding tissue is uncertain, particularly in a breast noted as heterogeneously dense.

**Figure 3:**
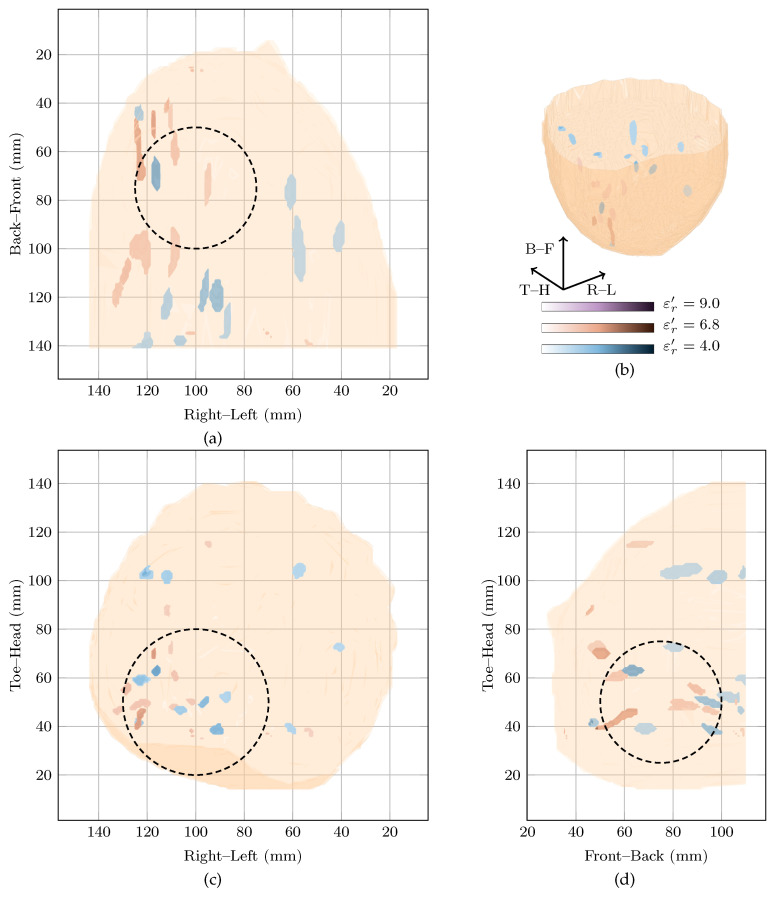
Images of high fitness and the image from the original study of Patient 2 compared in three dimensions in (**b**). Shown also are the coronal in (**d**), sagittal in (**c**) and axial in (**a**) slices.

The global maximum of the cost function in Figure 1 is located at $\varepsilon_{r}^{\prime }=4$ with a local maximum at $\varepsilon_{r}^{\prime }=6.8$. Both images are shown in Figure 3 in blue and red, respectively. The image reconstructed at the local maximum at $\varepsilon_{r}^{\prime }=6.8$ shows many responses in the lower inner quadrant, which is consistent with the clinical history of the patient reporting a fibroadenolipoma in that quadrant. However, the image is very difficult to interpret with multiple responses of similar magnitude. The image reconstructed at the global maximum at $\varepsilon_{r}^{\prime }=4$ also shows a lot of energy in the lower inner quadrant, but also some smaller responses elsewhere in the breast, including in the upper outer quadrant, although that response is 6 dB lower in amplitude than the main response in the image at $\varepsilon_{r}^{\prime }=4$. The image reconstructed at $\varepsilon_{r}^{\prime }=9.0$ is similar to the image at $\varepsilon_{r}^{\prime }=6.8$ but with lower overall amplitude (3 dB lower). Although the image reconstructed at $\varepsilon_{r}^{\prime }=9.0$ also contains a response in the lower inner quadrant which may correspond to the fibroadenolipoma, there is also many other responses with similar magnitude in the image.

Extremely dense breasts such as that of Patient 2 would be expected to have higher dielectric properties. However, the cost function rewards images reconstructed at lower dielectric properties very highly. Although it is difficult to draw any definite conclusions as the dielectric properties of the breast are not certain, it is likely the images rewarded by the cost function are reconstructed below the average dielectric properties of this particular breast and the images contain mostly spurious noise and clutter.

However, across the entire reconstruction permittivity range, no one image is characterised by one single response. As may be expected from a breast noted as extremely dense, all images contain many responses of similar magnitude. The poor image quality of this clinical case study may also be explained by a number of other factors, such as:

- Uncertain contrast between the fibroadenoma and the glandular and fibrous tissues in the rest of the breast;
- Difficulty in isolating reflections from the benign lesion from the reflections from the other glandular and fibrous structures in the breast;
- Acquisition challenges due to high attenuation in the dense breast tissues.

Due to these factors, it is difficult to predict what a “correct” radar-based image should look like for this clinical case study.

### 3.3. Invasive Ductal Carcinoma (Case 3)

The left breast of the 35-year-old Patient 3 was scanned. The mammogram indicated extensive microcalcifications around 3 o’clock in the lateral aspect and the magnetic resonance report showed enhancements from the 2 o’clock to 6 o’clock radian. Additionally, the magnetic resonance report showed a focal mass near the nipple. A region of invasive ductal carcinoma in the upper outer quadrant of the breast was reported after post-mastectomy pathology. The invasive ductal carcinoma was measured as 4 cm× 2 cm× 2 cm, although due to the location of the diseased tissue near the chest wall, it is uncertain how much of the disease was present within the imaging domain.

A prominent global maximum is present in the cost function at $\varepsilon_{r}^{\prime }=15.2$. This corresponds to an image with a single response located in the centre of the breast about 3 cm from the nipple. This response has an SMR of 44.98 dB and is nearly 11 dB larger in magnitude than the next highest response. This prominent response may correspond to the focal mass that was identified in the magnetic resonance image or, similar to Patient 2, this may be an artefact. In particular, due to the comparatively large reconstruction permittivity and the resulting large delay values, environmental noise, signals from longer propagation paths and errors in the artefact removal algorithms may be randomly cohering at this point in the centre of the imaging array. The minor peak at $\varepsilon_{r}^{\prime }=12.8$ also shows a response in the same location as the global maximum, but with an SMR of 40 dB.

**Figure 4:**
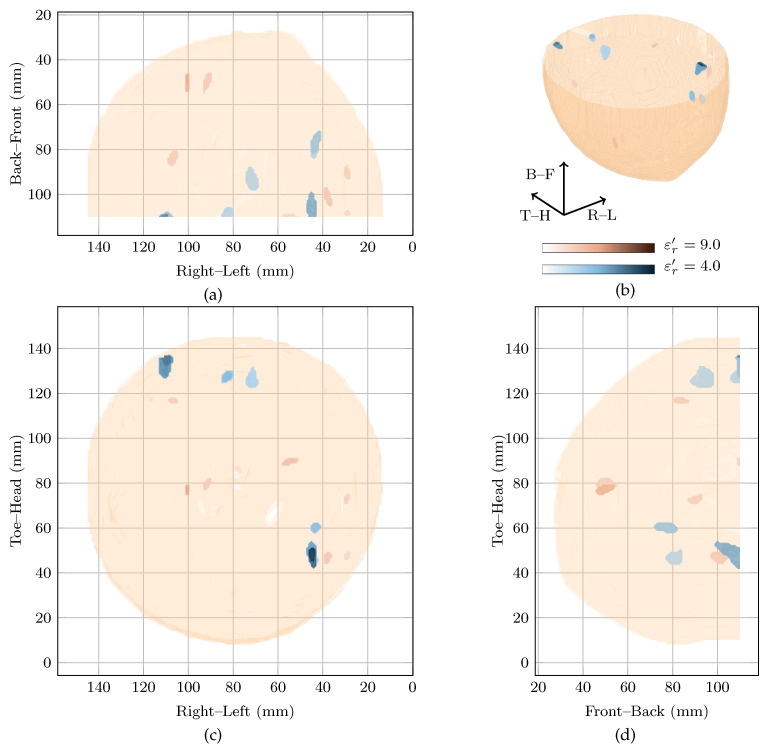
Images of high fitness and the image from the original study of Patient 3 compared in three dimensions in (**b**). Shown also are the coronal in (**d**), sagittal in (**c**) and axial in (**a**) slices.

The image in the original study, reconstructed at $\varepsilon_{r}^{\prime }=9$, is shown with the image at the local maximum at $\varepsilon_{r}^{\prime }=4$ in Figure 4. In the original image, the maximum response is located just above the nipple which could potentially correspond to the focal mass detected in the magnetic resonance image. In the image at the local maximum at $\varepsilon_{r}^{\prime }=4$, the maximum responses in the image are located towards the chest wall.

Although the breast contained extensive disease in this case study, no image clearly shows a response which could definitively be said to correspond to the invasive ductal carcinoma. The breast was noted as scattered heterogeneous according to the mammogram, meaning the average dielectric properties of the breast would be expected to be low. However, the image reconstructed at $\varepsilon_{r}^{\prime }=15.2$ is highly rewarded. Although this may correspond to a focal mass noted in the clinical history of the patient, it may also be an artefact due to reconstruction with overestimated dielectric properties.

### 3.4. Necrosis and Cysts (Case 4)

Patient 4 was 44 years old when her left breast was scanned with TSAR. Ultrasound and magnetic resonance imaging of the left breast showed an 11 mm × 7 mm lesion at the 10 o’clock radian which was determined as a fat necrosis from pathology. Two cysts were also reported near the fat necrosis. Similarly to the fibroadenolipoma for Patient 2, neither the exact contrast between the necrotic tissue and the surrounding breast is known, nor the exact locations where responses would be expected in the radar-based image.

**Figure 5:**
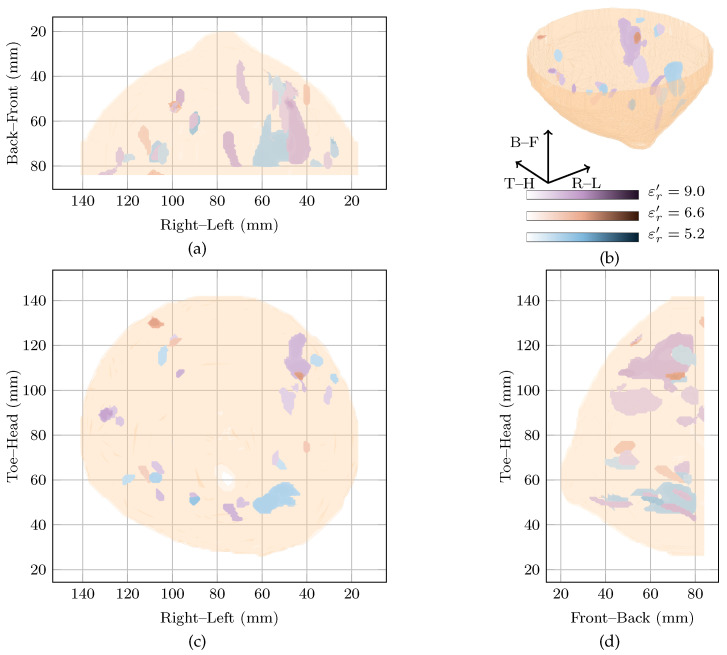
Images of high fitness and the image from the original study of Patient 4 compared in three dimensions in (**b**). Shown also are the coronal in (**d**), sagittal in (**c**) and axial in (**a**) slices.

The global maximum of the cost function is located at $\varepsilon_{r}^{\prime }=6.6$ with a local maximum at $\varepsilon_{r}^{\prime }=5.2$. These two images, along with the original image at $\varepsilon_{r}^{\prime }=9$ are shown in Figure 5. As can be seen, all three images contain many responses scattered throughout the breast.

Firstly, considering the global maximum at $\varepsilon_{r}^{\prime }=6.6$, the highest magnitude response is located in the upper outer quadrant. This response is nearly 5 dB higher than the next highest response and has an SMR of 25.61 dB. Two other responses located in the upper inner and the lower outer quadrants were within 5 dB of the highest amplitude in the image, and had SMRs of 20.69 dB and 20.26 dB, respectively.

Secondly, looking at the image at the local maximum at $\varepsilon_{r}^{\prime }=5.2$, the highest magnitude response is located in the lower inner quadrant. Similar to the global maximum at $\varepsilon_{r}^{\prime }=6.6$, a number of responses are visible in all four breast quadrants. For example, responses within 5 dB of the main response can be seen in the lower outer quadrant and the upper outer quadrant with SMRs of 18.74 dB and 18.36 dB compared to 21.62 dB for the main response.

Finally, comparing the images rewarded by the cost function to the image reconstructed at $\varepsilon_{r}^{\prime }=9$, all three images show responses in many quadrants of the breast. Due to the difficulties in reconciling the complex clinical history of the breast with the image, it is not clear if these responses correspond directly to any of the benign lesions in the breast.

### 3.5. No Breast Disease (Case 5)

Patient 5 was 32 years old when her left breast was scanned. The patient had no history of breast disease and the mammography report indicated the left breast was heterogeneous (BI-RADS C) with some glandular tissue both on the inner and outer sides of the breast. An initial magnetic resonance image suggested an unidentified lesion at 4 o’clock which was not apparent from mammography, a follow-up ultrasound or on a second magnetic resonance image.

**Figure 6:**
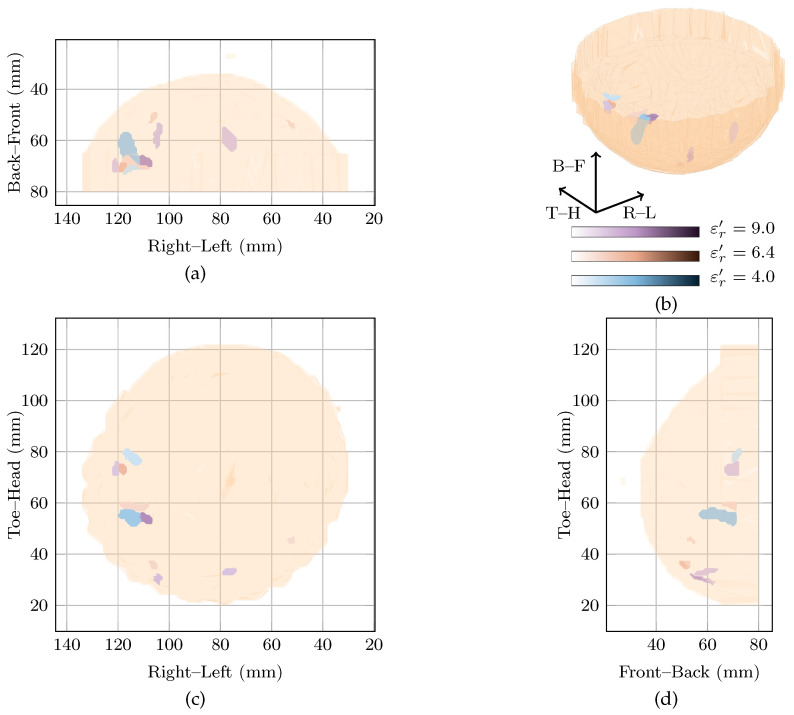
Images of high fitness and the image from the original study of Patient 5 compared in three dimensions in (**b**). Shown also are the coronal in (**d**), sagittal in (**c**) and axial in (**a**) slices.

The image at $\varepsilon_{r}^{\prime }=4$ is the most highly rewarded by the cost function, with a local maximum at $\varepsilon_{r}^{\prime }=6.4$. These images, as well as the image from the original study at $\varepsilon_{r}^{\prime }=9$ are shown in Figure 6. In all three images, responses on the inner side of the breast are visible close to the skin. These possibly correspond to fibroglandular tissue in these locations. In all the three images at increasing reconstruction permittivity, the SMRs were 21.77 dB, 26.28 dB and 28.2 dB and the main response was 6 dB, 4.89 dB and 5 dB higher than the next strongest response. In these images and the image in the original study, this case would likely be a false positive.

## 4. Conclusions

In this work, computational patient-specific microwave breast imaging is tested in five clinical case studies. The clinical case studies were obtained from the first-generation TSAR operational system developed at the University of Calgary and the microwave images were analysed using knowledge of the clinical history of the patient including recent mammograms, magnetic resonance images and pathology reports, where available. No definitive conclusions or trends can be drawn from only five clinical case studies, and in three of the five cases, a lot of uncertainty exists as to the dielectric composition of the breast and the location and extent of lesions in the patient breast. Despite these limitations, some interesting results were observed from the clinical case studies.

Firstly, in one case study (Case 1: the only case with known disease in a known location), reconstruction permittivity estimation rewards an image where a response corresponding to this tumour is identified. When compared to the fixed-value estimate used in the original study, the tumour response has a higher SMR, SCR and amplitude. This is consistent with the conclusions of the previous work in the literature, and may suggest that patient-specific beamforming could improve the sensitivity of the microwave breast imaging modality.

Secondly, it may be possible to reconstruct two images with the characteristics of correctly reconstructed images (tumour) within the relative permittivity range as seen in Patient 1. In this case, this may correspond to another benign lesion within the breast. It may be necessary to reconstruct multiple images with various reconstruction permittivity estimates to obtain a complete picture of the entire breast. At the very least, this clinical case study suggests that further testing in breasts with multiple regions of interest need to be investigated further.

Thirdly, these images are consistent with the hypothesis that obtaining high specificity with radar-based imaging may be difficult, but that patient-specific beamforming does not increase the false positives. For example, images of Patients 1 and 5 are visually very similar, even though one breast contains a tumour and the other does not, and it is likely that a radiologist review would identify suspicious regions in both images. However, it is interesting to note that the difference in maximum image magnitude between the diseased and healthy breast is 10.4 dB using the images selected by the cost function but only 1.6 dB for images selected in the original study. The maximum image magnitude is commonly identified as a means of distinguishing healthy and diseased breasts.

Finally, these cases studies are useful for identifying potential limitations of patient-specific beamforming. In many of these clinical cases, images reconstructed with very low reconstruction permittivity estimates are highly rewarded, including for a breast classed as extremely dense from mammography (contrary to expectations). It is difficult to understand why exactly this may be as the dielectric properties of those breasts are not well known, but this surprising result does suggest that if a very broad reconstruction permittivity search space is used, images with spurious responses may be reconstructed.

## Figures and Tables

**Table 1:** The clinical history of each case study.

Case	Age	Side	Density	Class	Abnormality
1	53	R	Heterogeneous	C	Metaplastic carcinoma
2	64	L	Extremely dense	D	Fibroadenolipoma
3	35	L	Scattered	B	Invasive ductal carcinoma
4	44	L	Heterogeneous	C	Necrosis and cysts
5	32	L	Heterogeneous	C	No abnormalities

**Table 2:** Images of high fitness and the image from the original study corresponding to Patient 1 are quantitatively compared, values are in (dB).

	$\varepsilon_{r}^{\prime }=5.4$	$\varepsilon_{r}^{\prime }=10.4$			$\varepsilon_{r}^{\prime }=9.0$		
	$M_{1}$	$M_{1}$	$M_{2}$	$M_{3}$	$M_{1}$	$M_{2}$	$M_{3}$
**Max.**	0	−1.65	−7.65	−7.84	−5.48	−6.10	−6.88
**Region**	$R_{1}^{1}$	$R_{2}^{1}$	$R_{3}^{1}$	$R_{1}^{1}$	$R_{1}^{1}$	$R_{3}^{1}$	$R_{2}^{1}$

## Data Availability

The data presented in this study are available on request from the corresponding author. The data are not publicly available due to privacy restrictions.
